# The psychosocial impact of flu influenza pandemics on healthcare workers and lessons learnt for the COVID-19 emergency: a rapid review

**DOI:** 10.1007/s00038-020-01463-7

**Published:** 2020-09-04

**Authors:** Serena Barello, Anna Falcó-Pegueroles, Debora Rosa, Angela Tolotti, Guendalina Graffigna, Loris Bonetti

**Affiliations:** 1 EngageMinds HUB – Consumer, Food and Health Engagement Research Center, L.go Gemelli 1, 20123 Milan, Italy; 2 Department of Psychology, Università Cattolica del Sacro Cuore, Milan, Italy; 3 Nursing Development and Research Unit, Oncology Institute of Southern Switzerland (IOSI), EOC, Via Ospedale, 1, 6500 Bellinzona, Switzerland; 4 Department of Fundamental Care and Medical-Surgical Nursing, School of Nursing, Faculty of Medicine and Health Sciences, Consolidated Research Group SGR 269 Quantitative Psychology, University of Barcelona, Barcelona, Spain; 5 Don Carlo Gnocchi Foundation, Milan, Italy; 6 Department of Business Economic Health and Social Care, University of Applied Sciences and Arts of Southern Switzerland, Manno, Switzerland

**Keywords:** COVID-19, Pandemics, Psychology, Health personnel, Nurse, Physician

## Abstract

**Objectives:**

During a pandemic, healthcare workers (HCWs) are essential to the health system response. Based on our knowledge, little information is available regarding the psychosocial impact on HCWs or interventions for supporting them during pandemics. Therefore, the study aimed to assess available literature on perceived stress and psychological responses to influenza pandemics in HCWs and identify implications for healthcare practice and future research.

**Methods:**

This is a rapid review of the literature. The review was conducted according to the Preferred Reporting Items for Systematic Review and Meta-Analysis.

**Results:**

Across all the studies—both qualitative and quantitative—HCWs working during the epidemic reported frequent concerns regarding their own health and the fear of infecting their families, friends and colleagues. Moreover, social isolation, uncertainty, fears of stigmatization and reluctance to work or considering absenteeism were frequently reported. Moreover, many studies highlighted a high prevalence of high levels of stress, anxiety and depression symptoms, which could have long-term psychological implications in HCWs.

**Conclusions:**

This rapid review offers an overview of the major concerns regarding HCWs’ psychosocial well-being and possible preventive strategies, which could be useful for the current COVID-19 outbreak and similar future pandemics. Studies suggested to invest on preventive psychological, social, family and physical support and to guaranteeing reasonable work conditions and others in order to protect HCWs from the long-lasting psychological effect of the COVID-19 pandemic.

**Electronic supplementary material:**

The online version of this article (10.1007/s00038-020-01463-7) contains supplementary material, which is available to authorized users.

## Introduction

The outbreak of COVID-19 has become a public health emergency of major international concern and has placed extraordinary demands upon healthcare systems worldwide.

At the time of preparing this manuscript (April 21, 2020), the World Health Organization (WHO) reported that there were 2,314,621 cases of infection across 213 countries (WHO 2020).

This health emergency is trigging an intense, international healthcare response, with thousands of healthcare workers (HCWs) at the frontline caring for those affected by the virus (Lai et al. 2020; Tan et al. 2020; The Lancet 2020) and is expected to cause enduring substantial physical, personal and emotional distress to healthcare providers providing direct care to COVID-19 patients (Lamiani et al. 2012; Falcó-Pegueroles et al. 2016; Lusignani et al. 2017; Delfrate et al. 2018; Lazzari et al. 2020).

Despite extensive epidemiological literature (Peeri et al. 2020) dealing with professionals’ burnout when dealing with infectious diseases and increasing research on interventions to support them during flu pandemics (Barello and Graffigna 2020; Barello et al. 2020a, b; Galbraith et al. 2020), to the best of our knowledge, there is a lack of systematization of studies conducted in this field.

Therefore, in this rapid review, we summarized the literature examining the psychosocial outcomes among HCWs involved in the management of flu pandemics. We used the results of this review to identify recommendations for interventions aimed at reducing the risk of adverse mental health outcomes and foster post-incident resilience within healthcare systems that may be affected by pandemics, like COVID-19.

## Methods

This rapid systematic review was conducted according to the Preferred Reporting Items for Systematic Review and Meta-Analysis (PRISMA) guidelines (Moher et al. 2009). Rapid reviews follow the principles of knowledge synthesis, including a clear statement of the objectives, eligibility criteria and the systematic presentation and synthesis of results. Therefore, the methods of a rapid review are similar to those of a systematic review, but the process adopted does not require the depth and breadth of a full systematic review (Tricco and Langlois 2017; Langlois et al. 2019). Rapid reviews have proved to be an efficient way to help policy-makers take informed decisions based on high-quality evidence generated in a timely manner.

### Study inclusion and exclusion criteria

All peer-reviewed research articles, written in English, Spanish and Italian, which focused on the effects on perceived stress or psychological responses or psychosocial functioning or mood status in HCWs providing direct patient care during an influenza pandemic outbreak were included. We included studies about all HCWs, with no distinction. To be consistent with today’s situation, we considered studies about influenza pandemic outbreaks similar to the COVID-2019 outbreak, such as the severe acute respiratory syndrome (SARS), the Middle East respiratory syndrome (MERS) and the swine flu pandemic (H1N1).

No restrictions were applied with regard to the designs of the eligible studies or on time of publication. The first search was conducted on 13 March 2020, and the last search was conducted on 24 April 2020. Situational reports, activity reports, conference reports/abstracts/summaries, letters to the editor and viewpoints were not included in this review. We searched the PubMed, CINAHL, PsycINFO and SCOPUS databases. All the search strategies are reported in Electronic Supplementary Material (ESM1).

### Study selection

The selection process was initially performed by SB and LB, by reading the titles and the abstracts. The full texts of the papers that met the inclusion criteria were then read by SB and LB independently, and finally, the decision to include or exclude a paper was reached jointly following a discussion. In case of disagreement, a third researcher (AFP) was involved. The study selection process is reported in Fig. 1 through the PRISMA flow chart.

### Data extraction

The data of the included studies were extracted by SB and LB independently and checked for consistency by the other authors (AFP, AT, DR, GG) in a systematic way. The following data were extracted from the studies: author(s) and year of publication, study site, work setting, sample, HCWs involved (target), study design and methods, type of pandemic disease, time of data collection since the beginning of the pandemic, outcome/measure explored, instruments, key findings and implications for practice.

### Synthesis of the results

We performed a narrative synthesis of the evidence of the included studies, according to the definition by Popay et al. (2006). First, we performed a description of the key findings of the included studies. Then, we organized the findings to map the evidence and synthesize the results of the included papers and to explore possible patterns.

## Results

### Search outcomes

The search of the electronic literature yielded 1055 unique citations. Ten citations were found through other sources. After removing the duplicates, 691 articles were assessed through the title and abstract and 621 were excluded. After reviewing the full texts, 36 articles were included in the review (see Fig. 1 in ESM2).

The key characteristics of the included studies are presented in Table 1. An exhaustive description of the studies’ main findings is reported in ESM3.

**Table 1:** Key characteristics of the included papers

First author, year	Country, CITY	Setting, TYPE of outbreak	Sample size and characteristics	Study design
*Quantitative studies*				
Austria-corrales et al. (2011)	Mexico, Mexico City	Tertiary care hospital, AH1N1	99 medical residents	Cross-sectional
Bai et al. (2004)	Taiwan	Psychiatric teaching hospital, SARS	338 HCWs/hospital staff members *(HCWs *= *218 and administrative personnel *= *79)*	Cross-sectional
Bukhari et al. (2016)	Saudi Arabia	N/A, MERS	386 Nurses	Cross-sectional
Chan and chan (2004)	Singapore	Tertiary care hospital, SARS	166 HCWs *(40 physicians and 137 nurses)*	Cross-sectional
Chen et al. (2005a)	Taiwan	COMMUNITY hospital, SARS	128 nurses *(42 low-risk units, 65 high-risk units and 21 forced to work in high-risk units)*	Cross-sectional
Chen et al. (2005b)	Taiwan	Emergency department, SARS	82 HCWs *(34 emergency physicians and 48 emergency nurses)*	Cross-sectional
Chong et al. (2004)	Taiwan	Tertiary care hospital, SARS	1257 healthcare workers *(nurses *= *676; doctors *= *139; health administrative workers *= *140; and other professionals*—*pharmacists, technicians and respiratory therapists*=* 302)*	Cross-sectional
Chua et al. (2004)	Hong Kong	SARS units	613 HCWs *(271 HCWs from SARS units and 342 healthy control subject)*	Cross-sectional
Goulia et al. (2010)	Greece, Ioánnina	University General Hospital, AH1N1	469 HCWs *(nurses *=* 209, physicians *=* 120, allied health workers *=* 59 and auxiliary health workers *=* 81)*	Cross-sectional
Grace et al. (2005)	Canada, Toronto	General teaching hospital, SARS	553 physicians	Cross-sectional
Khalid et al. (2016)	Saudi Arabia	Tertiary care hospital, MERS	117 HCWs *(nurses *= *89; physicians *= *16; and respiratory therapists *= *12)*	Cross-sectional
Lai et al. (2020)	China, multi-site	COVID-19 hospitals, COVID-19	1257 HCWs *(nurses *= *764 and physicians *=* 493)*	Cross-sectional
Lee et al. (2005)	Taiwan	General hospital, SARS	26 nurses	Cross-sectional
Lu et al. (2006)	Taiwan	Teaching hospital, SARS	127 HCWs *(physicians *=* 24; nurses *=* 49; and other hospital healthcare workers *=* 54)*	Cross-sectional
Lung et al. (2009) *(follow*-*up lu* et al*. (*2006*) study)*	Taiwan	Teaching hospital, SARS	127 HCWs *(physicians *=* 24; nurses *=* 49; and other hospital healthcare workers *=* 54*	Cross-sectional
Matsuishi et al. (2012)	Japan, Kobe	Three core General hospital, AH1N1	1625 HCWs *(physicians *=* 218; nurses *=* 864; and other members of staff *=* 543)*	Cross-sectional
Maunder et al. (2004) [follow-up of the (2003) study]	Canada, Toronto	2 university teaching hospitals and 1 health sciences centre for psychiatric illness and substance abuse, SARS	997 HCWs *(nurses *=* 430; clerical staff *=* 117; research laboratory and clinical laboratory staff *=* 223; physicians *=* 115; and administrator *= *112)*	Cross-sectional
Maunder et al. (2006) [follow-up of the (2004) study]	Canada, Toronto	9 Toronto SARS hospitals and 4 Hamilton non-SARS hospitals	Healthcare workers: survey A: 7*69 HCWs, nurses (565), clerical staff (64), physicians (22) and respiratory therapists (17). 99 HCWs were distributed among 14 other different job types* Survey B 187 HCWs *(Professions proportions were similar to the Survey A)*	Cross-sectional
Oh et al. (2017)	South Korea	5 Local Public Hospital, MERS	313 nurses *(participants classified according to their outbreak nursing experience.* ***First*-*hand group:*** *who provided direct care or screening for infected or suspected patients.* ***Second*-*hand group:*** *who provided care to general population with no suspected MERS symptoms*	Cross-sectional
Phua et al. (2005)	Singapore	Acute general hospital. Emergency Department of the national SARS screening centre in Singapore, SARS	96 HCWs *(physicians *=* 38 and nurses *=* 58)*	Cross-sectional
Sin and Huak (2004)	Singapore	Rehabilitative services department of a general hospital in Singapore, SARS	47 HCWs *(physiotherapists *=* 18; occupational therapists *=* 13; speech therapists *= *3; and support staff *=* 13)*	Cross-sectional
Styra et al. (2008)	Canada, Toronto	Toronto tertiary care healthcare institution, SARS	244 HCWs *(healthcare workers who work in high*-*risk areas *=* 160 and healthcare workers who work in low*-*risk areas *=* 84)*	Cross-sectional
Su et al. (2007)	Taiwan	Veterans General Hospital, SARS	105 nurses *SARS units *=* 75; non*-*SARS units *=* 32)*	Prospective and periodic follow-up design study.
Tam et al. (2004)	Hong Kong	3 hospitals (medical units and intensive care units), SARS	652 HCWs *(nurses *=* 404; healthcare assistants *=* 157; medical professionals *= *20; and other HCWs*—*occupational therapist and physiotherapist*=* 71)*	Cross-sectional
Tan et al. (2020)	Singapore	Tertiary care hospital COVID-19	470 HCWs *(medical healthcare personnel *=* 296; nurses *= *161; and physicians *= *135); non-medical healthcare personnel *=* 174 (allied healthcare professionals *= *65; technicians *= *10; clerical staff *= *30; administrator *= *33; and maintenance workers *= *36)*	Cross-sectional
Tham et al. (2005)	Singapore	Urban acute general hospital (medical units and intensive care units), SARS	96 HCWs *(physicians *=* 38 and nurses *=* 58)*	Cross-sectional
Tolomiczenko et al. (2005)	Canada, Toronto	Acute care facility—community hospital, SARS	300 HCWs *(registered nurses *=* 76, physicians *=* 62, manager *=* 29, other health professionals (occupational therapist, physiotherapist and speech*–*language pathologist) *=* 51 and other professions *=* 82)*	Cross-sectional
Verma et al. (2004)	Singapore	N/A, SARS	1050 HCWs *(GPs *= *721; traditional Chinese medicine practitioners *=* 329)*	Cross-sectional
Wong et al. (2005)	Hong Kong	Public hospitals (emergency departments), SARS	466 HCWS *(doctors, nurses and healthcare assistants)*	Cross-sectional
Wu et al. (2009)	Beijing, China	General Hospital, SARS	549 HCWs (doctors = 109; nurses = 206; technicians = 121; and others = 113)	Cross-sectional
*Qualitative studies*				
Almutairi et al. (2018)	Saudi Arabia,	Tertiary care hospital, MERS	7 HCWs *(nurses *= *4 and physicians *=* 3)*	Qualitative design
Corley et al. (2010)	Australia, Queensland	ICU, AH1N1	32 HCWs *(nurses *=* 28 and physicians *=* 4)*	Qualitative design
Holroyd and McNaught (2008)	Hong Kong	University of Hong Kong, SARS	7 senior nurses, attending master degree	Qualitative design
Khee et al. (2004)	Republic of Singapore	General hospital, SARS	188 HCWs	Qualitative design
Kim (2018)	Republic of Korea	Nurses from different care settings, MERS	12 nurses	Qualitative design
Maunder et al. (2003)	Canada, Toronto	University teaching hospital, SARS	HCWs *(profession not stated. There were also no demographic data)*	Qualitative design

HCWs, healthcare workers; SARS, *Severe acute respiratory syndrome;* MERS, *Middle East respiratory syndrome*; AH1N1, influenza A virus subtype H1N1; COVID-19, severe acute respiratory syndrome coronavirus 2

### Study characteristics

The 36 included studies were conducted in ten different countries, mainly Taiwan (*N* = 8, 22.2%), Republic of Singapore (*N* = 7, 19.4%), and Canada and China (*N* = 6, 16.7%). The vast majority of the studies had a cross-sectional design (*N* = 29, 80.5%) and were published between 2004 and 2020. Six studies had a qualitative design and one a prospective design. Self-reported questionnaires were used in all the cross-sectional studies. Most of the research studies were conducted during or soon after the pandemic (*N* = 23, 64%). More than half of the studies (*n*  =  25, 69.4%) regarded the 2003 SARS epidemic. Four studies were conducted during or immediately after the N1H1 outbreak, five studies after the MERS outbreak and two studies during the COVID-19 pandemic.

### Study participants and settings

The quantitative studies assessed a total of 13,711 participants. The number of respondents ranged between 26 and 1625. The qualitative studies involved a total of 246 participants, ranging from seven to 188. In most studies, female respondents were over-represented. The most represented clinical setting was the general teaching hospital (15 studies, 41.7%), followed by tertiary care hospitals (six studies, 16.7%). Nurses and physicians were the two types of HCWs mostly involved, with 28 (77.8%) and 23 (63.9%) studies, respectively, followed by healthcare assistants (HCAs). In 11 studies, the profession of the participant was not reported (see ESM4).

In cross-sectional studies, the response rate, when reported, varied between 27 and 96.9%. Cross-sectional studies usually examined the prevalence and correlates of epidemic-related psychosocial outcomes in several different HCW groups.

### Key findings

#### *Measurement of psychosocial outcomes*

Of the 36 studies, 20 adopted validated measures of psychosocial outcomes (Table 2). Five studies measured work-related stress and burnout, 16 measured post-traumatic stress disorder symptoms and 15 measured psychological well-being. Of the burnout studies, the majority used some variants of the *Maslach Burnout Inventory* (Maunder et al. 2006; Austria-Corrales et al. 2011). Post-traumatic stress disorder symptoms were mostly assessed (Chan and Chan 2004; Verma et al. 2004; Chong et al. 2004; Maunder et al. 2004, 2006; Sin and Huak 2004; Tham et al. 2005; Chen et al. 2005a, b; Phua et al. 2005; Styra et al. 2008; Wu et al. 2009; Matsuishi et al. 2012; Bukhari et al. 2016; Tan et al. 2020) with the *Impact of Event Scale* or some of its variants. The psychological well-being measures—adopted in 15 studies—were far more varied and in most of the studies included: the *General Health Questionnaire* (GHQ)—or a variant of it—which was used in seven studies (Chan and Chan 2004; Sin and Huak 2004; Tam et al. 2004; Verma et al. 2004; Phua et al. 2005; Tham et al. 2005; Goulia et al. 2010) or the *Chinese Health Questionnaire*, which was used in 3 studies.(Chong et al. 2004; Lu et al. 2006; Lung et al. 2009) Finally, mood symptoms were assessed though a wide range of instruments such as the *Beck Depression Inventory*, the *9*-*item Patient Health Questionnaire*, the *7*-*item Generalized Anxiety Disorder* and the *7*-*item Insomnia Severity Index*. Table 2 shows in more detail the specific instruments adopted by each study to measure the HCWs’ psychosocial outcomes related to the management of the epidemic. Few studies analysed psychological stress in non-clinical healthcare workers, such as administrative staff, clerical staff, logistic and maintenance staff. Although this staff is not directly involved in the care of patients, their work is of vital importance to sustain those in the front line. A study (Tan et al. 2020) reported that this staff had an even higher psychological distress than HCWs.

**Table 2:** A summary of the psychosocial variables, instruments adopted in the included studies and factors significantly related to each variable

Psychosocial outcome observed	Measuring instruments	Applied in	Factors significantly related to negative psychosocial outcomes			
			Sociodemographic and psychological characteristics	Professional characteristics	Organizational characteristics	Other characteristics
Work-related stress and burnout	*Maslach Burnout Inventory*	Austria-Corrales et al. (2011) Maunder et al. (2006)	*Duration of perceived risk* *Maladapting coping style (avoidance, hostile confrontation and self*-*blame)* *Poorer self*-*rated physical health condition* *Degree of worry about infection management* *Worries about spreading infection to colleagues, family and friends*	*Clinical specialization* *Job title* *Length of work experience* *Previous experiences with epidemics’ management* *Work satisfaction* *Lack of knowledge of the virus* *Not feeling trained about pandemics*	*Health status of infected colleagues* *Work setting (i.e. working in high-risk units)* *Unprotected exposure to infected patients* *Unpredictability of infection control measure*	*Being quarantined*
	*Korean Neuropsychiatric Association job stress questionnaire*	Oh et al. (2017)				
	*“MERSCoV staff questionnaire”*	Khalid et al. (2016)				
	*SARS Team Questionnaire*	Lee et al. (2005)				
Post-traumatic stress disorder (PTSD) symptoms	*Impact of Event Scale* *Or* *22*-*item Impact of Event Scale*–*Revised*	Bukhari et al. (2016) Chan and Chan (2004) Chen et al. (2005a) Chong et al. (2004) Matsuishi et al. (2012) Maunder et al. (2004) Maunder et al. (2006) Phua et al. (2005) Sin and Huak (2004) Styra et al. (2008) Tham et al. (2005) Tan et al. (2020) Verma et al. (2004) Wu et al. (2009)	*Gender* *Age* *Availability of social support* *Depressive affect* *Perceived impact on personal life* *Duration of perceived risk of infection* *Maladapting coping style (avoidance, hostile confrontation and self*-*blame)* *Sense of control* *Perceived negative feelings towards the epidemic* *Previous history of mood disorders*	*Length of work experience* *Job title* *Not feeling trained about pandemics*	*Lack of clear communication from organizations* *Lack of colleagues’ support* *Specific clinical procedures (i.e. emergency resuscitation)* *Unprotected exposure to infected patients* *Work load* *Work setting (i.e. working in high-risk units or disease*-*specific units)*	–
	*Davidson trauma scale (DTS*-*C)*	Su et al. (2007)				
Psychological well-being	*General Health Questionnaire 28*	Chan and Chan (2004) Goulia et al. (2010) Phua et al. (2005) Sin and Huak (2004) Tam et al. (2004) Tham et al. (2005) Verma et al. (2004)	*Marital status* *Educational level* *Gender* *Age* *Maladapting coping style* *Perceived risk of self*-*infection* *Attachment style* *Be less willing to work in high-risk units* *Daily*-*life stressful events* *Personality traits* *Poor self*-*rated physical health* *Attachment anxiety* *Confidence about infection control* *Previous history of mood disorders*	*Job title* *Work satisfaction* *Higher levels of job-related stress* *Technical title (i.e. junior, intermediate, senior)*	*Lack of clear communication from organizations* *Lack of colleagues’ support* *Unprotected exposure to infected patients* *Inadequate support* *Unprotected exposure to infected patients* *Work setting (i.e. working in high-risk units)* *Type of hospital (tertiary vs secondary hospital)* *Working in “red zone” areas* *Frontline healthcare workers*	*Phase of the epidemic course (i.e. “the repair phase”)* *Being quarantined*
	*Chinese Health Questionnaire*	Chong et al. (2004) Lu et al. (2006) Tam et al. (2004)				
	*Kessler Psychological Distress Scale*	Maunder et al. (2004)				
	*Beck depression inventory*	Su et al. (2007)				
	*Depression, Anxiety and Stress Scales*	Tan et al. (2020)				
	*Perceived Stress Scale*	Chua et al. (2004)				
	*SCL*-*90R*	Chen et al. (2005a)				
	*9*-*item Patient Health Questionnaire*	Lai et al. (2020)				
	*7*-*item generalized anxiety disorder*	Lai et al. (2020)				
	*7*-*item Insomnia severity index*	Lai et al. (2020)				
	*Pittsburgh sleep quality index*	Su et al. (2007)				
	*Spielberger trait anxiety inventory*	Su et al. (2007)				

#### *Impact findings about the psychosocial response to pandemics*

Across all the studies—both qualitative and quantitative—HCWs working during the epidemic reported frequent concerns regarding their own health and the fear of infecting their families, friends and colleagues. They frequently suffered social isolation (Maunder et al. 2003, 2004, 2006), uncertainty (Chong et al. 2004) and fears of stigmatization (Bai et al. 2004; Verma et al. 2004), reluctance to work or considering absenteeism (Bai et al. 2004). Moreover, many studies highlighted a high prevalence of high levels of stress, anxiety and depression symptoms, which could have long-term psychological implications in HCWs (Maunder et al. 2003; Chong et al. 2004; Chen et al. 2005a; Grace et al. 2005; Su et al. 2007; Matsuishi et al. 2012; Lai et al. 2020). Stigmatization was a frequent theme emerging also in qualitative studies (Maunder et al. 2003; Almutairi et al. 2018).

#### *Factors associated with the psychosocial response to pandemics*

Four main categories of variables related to psychosocial outcomes were identified: (1) sociodemographics; (2) psychological characteristics; (3) professional attitudes and characteristics; and (4) organizational environment. Finally, two contextual elements appeared relevant in shaping the psychological reactions of HCWs: being quarantined and the epidemiological phase of the disease outbreak.

#### *Sociodemographics*

Among the sociodemographic factors, age (Wu et al. 2009), sex (Chong et al. 2004; Lai et al. 2020), marital status (Chen et al. 2005a, b) and educational level (Chua et al. 2004) showed some associations with epidemic-related psychosocial outcomes on HCWs, although circumstantial.

#### *Organizational aspects*

Several studies reported the relationship between HCWs’ psychosocial outcomes and organizational aspects, such as working in high-risk locations (Chua et al. 2004; Chen et al. 2005a; Styra et al. 2008), lack of clear communication from organizations (Chan and Chan 2004), lack of support from colleagues (Chan and Chan 2004), specific clinical procedures (i.e. emergency resuscitation) (Chen et al. 2005b), unprotected exposure to infected patients (Lu et al. 2006; Styra et al. 2008) and inadequate organizational support (i.e. counselling and psychological support from the employer, and insurance and compensation)(Khalid et al. 2016).

#### *Professional characteristics*

Some studies reported the relationship between psychosocial distress and professional characteristics, such as job titles (Chen et al. 2005a), work satisfaction (Tolomiczenko et al. 2005), job-related stress (Maunder et al. 2004), technical titles (i.e. junior, intermediate, senior) (Chen et al. 2005a, b; Khalid et al. 2016) and not feeling sufficiently trained in infection management (Wong et al. 2005).

#### *Personality characteristics*

Some studies focused on the relationship between psychosocial distress and individual psychological resources or characteristics, such as maladapting coping style (Chan and Chan 2004; Maunder et al. 2006; Oh et al. 2017), social isolation (Maunder et al. 2004; Goulia et al. 2010), perceived risk of self-infection (Khalid et al. 2016), previous history of mood disorders (Su et al. 2007), personality traits (Lu et al. 2006) and attachment style (Lu et al. 2006; Lung et al. 2009).

Finally, across the factors associated with the psychosocial outcomes, the specific phase of the epidemic course has been shown to be associated with symptom exacerbation (Wu et al. 2009).

### Preventive strategies

A wide range of intervention strategies to reduce emotional distress in HCWs exposed to the epidemic outbreaks emerged from the included studies, which can be classified in policy, organizational and person-directed strategies (see ESM5 for a detailed synthesis).

At the policy level, nine studies suggested to develop a strategic plan for future outbreaks (Sin and Huak 2004; Tolomiczenko et al. 2005; Wong et al. 2005; Lu et al. 2006; Maunder et al. 2006; Holroyd and McNaught 2008; Lung et al. 2009; Corley et al. 2010; Kim 2018) and one study to conduct public campaigns to protect HCWs and reduce their stigmatization (Matsuishi et al. 2012). From an organizational point of view, many studies underlined how it is important to ensure favourable work conditions (Bai et al. 2004; Maunder et al. 2006; Su et al. 2007; Austria-Corrales et al. 2011; Matsuishi et al. 2012) and provide HCWs with all the personal protective equipment (PPE) necessary to work safely and reduce their risk (Chen et al. 2005a, b; Goulia et al. 2010; Khalid et al. 2016). Organizations should also promote HCWs personal coping strategies, such as altruism, acceptance, resilience and humour (Lee et al. 2005; Wong et al. 2005; Wu et al. 2009).

The majority of the included studies underlined the importance of psychological support before, during and after the outbreak, provided by specially trained personnel (Bai et al. 2004; Tam et al. 2004; Verma et al. 2004; Chan and Chan 2004; Chong et al. 2004; Khee et al. 2004; Lee et al. 2005; Phua et al. 2005; Tham et al. 2005; Wong et al. 2005; Chen et al. 2005a, b; Grace et al. 2005; Su et al. 2007; Styra et al. 2008; Wu et al. 2009; Corley et al. 2010; Matsuishi et al. 2012; Almutairi et al. 2018; Kim 2018; Lai et al. 2020; Tan et al. 2020). It is important also to provide social support for HCWs’ families (Grace et al. 2005; Bukhari et al. 2016) and recognize HCWs’ efforts by providing positive feedback (Maunder et al. 2006; Khalid et al. 2016). Included studies highlighted also how physical well-being is important to maintain psychological stability (Maunder et al. 2003; Bai et al. 2004; Goulia et al. 2010). A collaborative climate within the clinical team is also important to promote social support, and reduce conflict and the negative effects of social isolation (Maunder et al. 2003, 2004, 2006; Khee et al. 2004; Lee et al. 2005). Furthermore, studies—both qualitative and quantitative—stressed the centrality of providing HCWs with accurate and timely information to reduce uncertainty (Maunder et al. 2003, 2004, 2006; Bai et al. 2004; Sin and Huak 2004; Corley et al. 2010; Goulia et al. 2010; Matsuishi et al. 2012; Khalid et al. 2016) as well as training and education about how to protect themselves and properly deal with infected patients (Maunder et al. 2003, 2004, 2006; Bai et al. 2004; Sin and Huak 2004; Chua et al. 2004; Chen et al. 2005a, b; Corley et al. 2010; Bukhari et al. 2016; Oh et al. 2017).

## Discussion

This rapid review included 36 studies addressing the psychosocial outcomes among HCWs working during pandemics. Across the studies, there was evidence showing how during critical situations like flu pandemics, HCWs are at risk of developing psychological distress. Moreover, many risk factors are reported to impact on psychological outcome. Such factors are related to sociodemographic, organizational and individual professionals’ characteristics. Some preliminary studies about COVID-19—which are included in this review—are just demonstrating the immediate impact of such emergency on professionals’ health.

In the next paragraph, a synthesis of the main recommendations to mitigate the effects of pandemics on professionals’ well-being is provided.

### What can be done to reduce the psychosocial distress in healthcare workers during pandemics?

The studies included reported very similar strategies to improve the overall health system’s reaction to such crisis, confirming that some interventions have already proved to be useful in this regard.

### Policy-related strategies

Firstly, it is important that the whole national health system is involved in the development of preventive strategies (Sin and Huak 2004; Tolomiczenko et al. 2005; Wong et al. 2005; Lu et al. 2006; Maunder et al. 2006; Holroyd and McNaught 2008; Lung et al. 2009; Corley et al. 2010; Kim 2018). Corley et al. (2010) underlined the relevance to plan a strategic approach for future pandemics, through effective information regarding infection control interventions, in both clinical and non-clinical settings. Corley et al. (2010) underlined also the importance to have an adequate staff requirement plan in advance, in order to be prepared when an outbreak starts and stressed the importance of planning appropriate training for HCWs. Wong et al. (2005) underlined the importance of planning ahead of time proactive psychological support. Matsuishi et al. (2012) suggested to develop public campaigns to protect HCWs and reduce stigmatization. Because the risk of other pandemic outbreaks will probably increase in the future, Tolomiczenko et al. (2005) stressed the importance of maintaining high levels of vigilance.

### Organization-related strategies

Hospital and primary care organizations have an extremely important role in the prevention of psychosocial stress in HCWs. It is necessary to guarantee favourable work condition (Bai et al. 2004; Chen et al. 2005a, b; Maunder et al. 2006; Su et al. 2007; Goulia et al. 2010; Austria-Corrales et al. 2011; Matsuishi et al. 2012; Khalid et al. 2016). Ensuring adequate staffing levels to guarantee the necessary rest for HCWs is mandatory to maintain their psychological and physical well-being (Bai et al. 2004; Maunder et al. 2006; Goulia et al. 2010; Austria-Corrales et al. 2011). In this regard, Maunder et al. (2006) suggested that an appropriate nurse–patient ratio, also in normal conditions, is mandatory for the future. The importance of personal protective equipments (PPEs) has been stressed in many studies (Chen et al. 2005a, b; Goulia et al. 2010; Khalid et al. 2016). They reduce HCWs’ fear of self-infection or of infecting their relatives and patients and therefore promote a less stressful approach to the clinical practice. During the current COVID-19 outbreak, this was one of the major issues, mostly in the western countries, where the production of masks was stopped for economic reason in recent years. This has caused the lack of masks and consequently an increased number of infections and deaths among HCWs (The Lancet 2020).

As reported by Maunder et al. (2006), the pre-pandemic period is a critical time during which organizations should address their weaknesses by recruiting sufficient staff, increasing nurses’ autonomy, control over practice, flexibility and perceived empowerment. This has been a particularly critical aspect during COVID-19 outbreak, because many health institutions were unprepared from an organizational point of view.

### Person-directed strategies

#### *Providing accurate and timely information and training*

First, the article underlined the importance of an accurate information about the disease spread mechanisms, so as to give the opportunity to HCWs to protect themselves and their families (Maunder et al. 2003, 2004, 2006; Bai et al. 2004; Sin and Huak 2004; Corley et al. 2010; Goulia et al. 2010; Matsuishi et al. 2012; Khalid et al. 2016). Therefore, health authorities, such as the World Health Organization (WHO), must to be very clear about infection control matters and how to deal with infected patients. This can reduce HCWs’ fear and sense of inadequacy, aspects that can increase psychological distress. Bai et al. (2004) stated that adequate information among the population can also reduce HCW stigmatization. Sin and Huak (2004) underlined the importance of having good communication channels and efficient information dissemination, not only for the public, but also for healthcare facilities, to ensure a more efficient and effective approach towards the emergency. This is one of the major issues of the current COVID-19 pandemic. From the beginning, the media were giving contradictory information. Worst of all, the large quantity of fake news spread even faster than the disease.

A second aspect stressed by the studies included in this review to reduce psychological distress was the importance of HCWs’ appropriate training about patient isolation procedures, use of PPE and recognizing symptoms (Maunder et al. 2003, 2004, 2006; Bai et al. 2004; Sin and Huak 2004; Chua et al. 2004; Chen et al. 2005a, b; Corley et al. 2010; Bukhari et al. 2016; Oh et al. 2017). Alike adequate information, also training can reduce HCWs’ fear and sense of inadequacy (Maunder et al. 2003, 2004, 2006; Bai et al. 2004; Sin and Huak 2004; Chua et al. 2004; Chen et al. 2005a, b; Corley et al. 2010; Bukhari et al. 2016; Oh et al. 2017). It can also increase their confidence in dealing with the pandemic. Also humour in the workplace should be promoted, because they can soothe the sense of fear and encourage teamwork (Lee et al. 2005). It is also important to promote altruism and resilience behaviours (Wong et al. 2005; Wu et al. 2009). Therefore, training on communication skills in emergency scenarios should be provided. Moreover, both Su et al. (2007) and Sin and Huak (2004) highlighted that novice HCWs have a higher risk of psychological distress, due to their inexperience. Although in emergency circumstances it is not easy to have adequate and prepared staff for all situations, it is important that hospital management should try to avoid putting novice HCWs in high-risk units, and give the priority to more experienced and trained staff (Chen et al. 2005a, b; Lee et al. 2005; Maunder et al. 2006; Oh et al. 2017).

#### Provide psychological, social, physical, ethical and family support to HCWs

The majority of the studies highlighted the importance of psychological support during and after a pandemic (Bai et al. 2004; Tam et al. 2004; Verma et al. 2004; Chan and Chan 2004; Chong et al. 2004; Khee et al. 2004; Lee et al. 2005; Phua et al. 2005; Tham et al. 2005; Wong et al. 2005; Chen et al. 2005a, b; Grace et al. 2005; Su et al. 2007; Styra et al. 2008; Wu et al. 2009; Corley et al. 2010; Matsuishi et al. 2012; Almutairi et al. 2018; Kim 2018; Lai et al. 2020; Tan et al. 2020). Khee et al. (2004) highlighted the usefulness of psychotherapeutic groups during the SARS outbreak in Singapore, because they constituted a source of mutual support for HCWs. As underlined by Lee et al. (2005), it is important also to provide psychiatric support for HCWs who work in high-risk environments. Phua et al. (2005) underlined that to ensure effective psychological support, it is important to know which coping strategies are being adopted by HCWs so that they can be promoted (Verma et al. 2004). Some studies reported about the importance of team climate (Maunder et al. 2003, 2004, 2006; Khee et al. 2004; Lee et al. 2005) because it can reduce the negative effects of social isolation (Maunder et al. 2003, 2004, 2006). In fact, also during the COVID-19 outbreak many HCWs decided to isolate themselves to not infect their families (Lee et al. 2005; Fichtel and Kaufman 2020).

Finally, it is important to sustain HCWs from a physical point of view (Maunder et al. 2003; Bai et al. 2004; Goulia et al. 2010). For instance, guaranteeing a restorative sleep may be the first aspect to consider because during these crises sleep deprivation and insomnia are frequent (Maunder et al. 2006a).

### Limitations

This review has strengths and limitations. Since this rapid review aimed to provide a timely overview of what happens during a pandemic and provide useful suggestions on how to deal with the current COVID-19 outbreak, no formal quality appraisal of the included studies was conducted. However, according to the guidelines of Grant and Booth (2009), we carefully built the research question by extracting only the key variables. Moreover, we only included peer-reviewed publications and did not consider any relevant grey literature. Another limitation is that the majority of the included studies had a cross-sectional design and a convenience sample.

### Conclusions

This rapid review gives some valuable suggestions for the analysis of pandemics’ outbreaks and its understanding in terms of its effects on the healthcare workforce well-being. The current COVID-19 pandemic caught many countries totally unprepared to deal with the emergency, especially the western ones. Since a second wave of COVID-19 cannot be excluded in the next months, findings from this study could be particularly useful also for the current pandemic.

## Electronic supplementary material

Below is the link to the electronic supplementary material.

Supplementary material 1 (DOCX 66 kb)

## References

[CR1] Almutairi AF, Adlan AA, Balkhy HH (2018). “It feels like I’m the dirtiest person in the world”: exploring the experiences of healthcare providers who survived MERS-CoV in Saudi Arabia. J Infect Public Health.

[CR2] Austria-Corrales F, Cruz-Valdés B, Herrera-Kiengelher L (2011). Síndrome de burnout en médicos mexicanos en entrenamiento durante una contingencia sanitaria por virus de influenza A H1N1. Gac Med Mex.

[CR3] Bai YM, Lin CC, Lin CY (2004). Survey of stress reactions among health care workers involved with the SARS outbreak. Psychiatr Serv.

[CR4] Barello S, Graffigna G (2020). Caring for Health Professionals in the COVID-19 Pandemic emergency: toward an “Epidemic of Empathy” in Healthcare. Front Psychol.

[CR5] Barello S, Palamenghi L, Graffigna G (2020). Burnout and somatic symptoms among frontline healthcare professionals at the peak of the Italian COVID-19 pandemic. Psychiatry Res.

[CR6] Barello S, Palamenghi L, Graffigna G (2020). Empathic communication as a “Risky Strength” for Health during the COVID-19 Pandemic: the Case of Frontline Italian Healthcare Workers. Patient Educ Couns.

[CR7] Bukhari EE, Temsah MH, Aleyadhy AA (2016). Middle east respiratory syndrome coronavirus (MERS-CoV) outbreak perceptions of risk and stress evaluation in nurses. J Infect Dev Ctries.

[CR8] Chan AOM, Chan YH (2004). Psychological impact of the 2003 severe acute respiratory syndrome outbreak on health care workers in a medium size regional general hospital in Singapore. Occup Med (Chic Ill).

[CR9] Chen CS, Wu HY, Yang P, Yen CF (2005). Psychological distress of nurses in Taiwan who worked during the outbreak of SARS. Psychiatr Serv.

[CR10] Chen WK, Cheng YC, Chung YT, Lin CC (2005). The impact of the SARS outbreak on an urban emergency department in Taiwan. Med Care.

[CR11] Chong MY, Wang WC, Hsieh WC (2004). Psychological impact of severe acute respiratory syndrome on health workers in a tertiary hospital. Br J Psychiatry.

[CR12] Chua SE, Cheung V, Cheung C (2004). Psychological effects of the SARS outbreak in Hong Kong on high-risk health care workers. Can J Psychiatry.

[CR13] Corley A, Hammond NE, Fraser JF (2010). The experiences of health care workers employed in an Australian intensive care unit during the H1N1 Influenza pandemic of 2009: a phenomenological study. Int J Nurs Stud.

[CR14] Delfrate F, Ferrara P, Spotti D (2018). Moral Distress (MD) and burnout in mental health nurses: a multicenter survey. Med del Lav..

[CR15] Falcó-Pegueroles A, Lluch-Canut MT, Martínez-Estalella G (2016). Levels of exposure to ethical conflict in the ICU: correlation between sociodemographic variables and the clinical environment. Intensive Crit Care Nurs.

[CR16] Fichtel C, Kaufman S (2020). Fearing COVID-19 spread to families, health care workers self-isolate at home.

[CR17] Galbraith N, Boyda D, McFeeters D, Hassan T (2020). The mental health of doctors during the COVID-19 pandemic. BJPsych Bull.

[CR18] Goulia P, Mantas C, Dimitroula D (2010). General hospital staff worries, perceived sufficiency of information and associated psychological distress during the A/H1N1 influenza pandemic. BMC Infect Dis.

[CR19] Grace SL, Hershenfield K, Robertson E, Stewart DE (2005). The occupational and psychosocial impact of SARS on academic physicians in three affected hospitals. Psychosomatics.

[CR20] Grant MJ, Booth A (2009). A typology of reviews: an analysis of 14 review types and associated methodologies. Health Info Libr J.

[CR22] Holroyd E, McNaught C (2008). The SARS crisis: reflections of Hong Kong nurses. Int Nurs Rev.

[CR23] Khalid I, Khalid TJ, Qabajah MR, Barnard AG, Qushmaq IA (2016). Healthcare workers emotions, perceived stressors and coping strategies during a MERS-CoV outbreak. Clin Med Res.

[CR24] Khee KS, Lee LB, Chai OT (2004). The psychological impact of SARS on health care providers. Crit Care Shock.

[CR25] Kim Y (2018). Nurses’ experiences of care for patients with Middle East respiratory syndrome-coronavirus in South Korea. Am J Infect Control.

[CR26] Lai J, Ma S, Wang Y (2020). Factors Associated With Mental Health Outcomes Among Health Care Workers Exposed to Coronavirus Disease 2019. JAMA Netw open.

[CR27] Lamiani G, Barello S, Browning DM (2012). Uncovering and validating clinicians’ experiential knowledge when facing difficult conversations: a cross-cultural perspective. Patient Educ Couns.

[CR28] The Lancet (2020). COVID-19: protecting health-care workers. Lancet.

[CR29] Langlois EV, Straus SE, Antony J (2019). Using rapid reviews to strengthen health policy and systems and progress towards universal health coverage. BMJ Glob Heal.

[CR30] Lazzari T, Terzoni S, Destrebecq A (2020). Moral distress in correctional nurses: a national survey. Nurs Ethics.

[CR31] Lee S-H, Juang Y-Y, Su Y-J (2005). Facing SARS: psychological impacts on SARS team nurses and psychiatric services in a Taiwan general hospital. Gen Hosp Psychiatry.

[CR32] Lu Y-C, Shu B-C, Chang Y-Y, Lung F-W (2006). The mental health of hospital workers dealing with severe acute respiratory syndrome. Psychother Psychosom.

[CR33] Lung F-W, Lu Y-C, Chang Y-Y, Shu B-C (2009). Mental symptoms in different health professionals during the SARS attack: a Follow-up study. Psychiatr Q.

[CR34] Lusignani M, Giannì ML, Re LG, Buffon ML (2017). Moral distress among nurses in medical, surgical and intensive-care units. J Nurs Manag.

[CR35] Matsuishi K, Kawazoe A, Imai H (2012). Psychological impact of the pandemic (H1N1) 2009 on general hospital workers in Kobe. Psychiatry Clin Neurosci.

[CR36] Maunder R, Hunter J, Vincent L (2003). The immediate psychological and occupational impact of the 2003 SARS outbreak in a teaching hospital. CMAJ.

[CR37] Maunder RG, Lancee WJ, Rourke S (2004). Factors associated with the psychological impact of severe acute respiratory syndrome on nurses and other hospital workers in Toronto. Psychosom Med.

[CR38] Maunder R, Lancee W, Balderson K (2006). Long-term psychological and occupational effects of providing hospital healthcare during SARS outbreak. Emerg Infect Dis.

[CR39] Moher D, Liberati A, Tetzlaff J (2009). Preferred reporting items for systematic reviews and meta-analyses: the PRISMA statement. PLoS Med.

[CR40] Oh N, Hong N, Ryu DH (2017). Exploring nursing intention, stress, and professionalism in response to infectious disease emergencies: the experience of local public hospital nurses during the 2015 MERS Outbreak in South Korea. Asian Nurs Res (Korean Soc Nurs Sci).

[CR41] Peeri NC, Shrestha N, Rahman MS (2020). The SARS, MERS and novel coronavirus (COVID-19) epidemics, the newest and biggest global health threats: what lessons have we learned?. Int J Epidemiol.

[CR42] Phua DH, Tang HK, Tham KY (2005). Coping responses of emergency physicians and nurses to the 2003 severe acute respiratory syndrome outbreak. Acad Emerg Med.

[CR43] Popay J, Roberts H, Sowden A et al (2006) Guidance on the conduct of narrative synthesis in systematic reviews. A Prod from ESRC methods Program Version 1:b92

[CR44] Sin SS, Huak CY (2004). Psychological impact of the SARS outbreak on a Singaporean rehabilitation department. Int J Ther Rehabil.

[CR45] Styra R, Hawryluck L, Robinson S (2008). Impact on health care workers employed in high-risk areas during the Toronto SARS outbreak. J Psychosom Res.

[CR46] Su TP, Lien TC, Yang CY (2007). Prevalence of psychiatric morbidity and psychological adaptation of the nurses in a structured SARS caring unit during outbreak: a prospective and periodic assessment study in Taiwan. J Psychiatr Res.

[CR47] Tam CWC, Pang EPF, Lam LCW, Chiu HFK (2004). Severe acute respiratory syndrome (SARS) in Hongkong in 2003: stress and psychological impact among frontline healthcare workers. Psychol Med.

[CR48] Tan B, Chew N, Lee G (2020). Psychological impact of the COVID-19 Pandemic on health care workers in Singapore. Ann Intern Med.

[CR49] Tham KY, Tan YH, Loh OH (2005). Psychological morbidity among emergency department doctors and nurses after the SARS outbreak. Hong Kong J Emerg Med.

[CR50] Tolomiczenko GS, Ricci M, Strathern L (2005). SARS: coping with the impact at a community hospital. J Adv Nurs.

[CR51] Tricco AC, Langlois EVSS (2017). Rapid reviews to strengthen health policy and systems: a practical guide.

[CR52] Verma S, Mythily S, Chan YH (2004). Post-SARS psychological morbidity and stigma among general practitioners and traditional Chinese medicine practitioners in Singapore. Ann Acad Med Singap.

[CR53] WHO (2020) Coronavirus disease 2019 (COVID-19) Situation Report-68

[CR54] Wong TW, Yau JKY, Chan CLW (2005). The psychological impact of severe acute respiratory syndrome outbreak on healthcare workers in emergency departments and how they cope. Eur J Emerg Med.

[CR55] Wu P, Fang Y, Guan Z (2009). The psychological impact of the SARS epidemic on hospital employees in China: exposure, risk perception, and altruistic acceptance of risk. Can J Psychiatry.

