# Supplementary material for: The psychosocial impact of flu influenza pandemics on healthcare workers and lessons learnt for the COVID-19 emergency: a rapid review
Source: Int J Public Health. 2020 Sep 4;65(7):1205–16. doi: 10.1007/s00038-020-01463-7 (PMC7472941; doi:10.1007/s00038-020-01463-7)
Supplement: Supplementary file 1 — Supplementary material 1 (DOCX 66 kb) [file 38_2020_1463_MOESM1_ESM.docx]

Journal International Journal of Public Health

Article Title: The Psycho-social Impact of Flu Influenza Pandemics on Healthcare Workers and lessons learnt for the COVID-19 emergency: A Rapid Review

**ECM 1. Search strategy’s strings (last view 24^th^ April 2020)**

To formulate the research question we used the PIO variant of the PICO (P=Patient, problem or population; I=Intervention; C=Comparison or Control; O=Outcome) framework because there was no comparison, control or comparator, as reported below:

- *Population:* healthcare workers
- *Intervention***:** working during an influenza pandemic outbreak similar to the COVID-2019 outbreak
- *Outcome:* effects on perceived stress and psychological responses or psychosocial functioning or mood status

**PUBMED:**

((((((("Disease Outbreaks"[Mesh]) OR "Influenza A Virus, H1N1 Subtype"[Mesh] OR "COVID-19" [Supplementary Concept]))) AND (("Health Knowledge, Attitudes, Practice"[Mesh]) OR "Attitude of Health Personnel"[Mesh]))) AND (("Allied Health Personnel"[Mesh]) OR ((("Nurses"[Mesh]) OR "Health Personnel"[Mesh]) OR "Physicians"[Mesh])))) AND (((("Adaptation, Psychological"[Mesh]) OR "Resilience, Psychological"[Mesh])) OR "Health Personnel/psychology"[Mesh])

**CINAHL:**

| S13 | (S4 OR S11) AND (S7 AND S10 AND S12) | (210) |
| --- | --- | --- |
| S12 | S4 OR S11 | (177,786) |
| S11 | (MH "Adaptation, Psychological+") OR "Resilience, Psychological" | (42,361) |
| S10 | S8 OR S9 | (29,277) |
| S9 | (MH "Disease Outbreaks") | (28,311) |
| S8 | (MH "Coronavirus Infections") OR (MH "Middle East Respiratory Syndrome Coronavirus") OR (MH "SARS Virus") OR (MH "Middle East Respiratory Syndrome") OR (MH "Coronavirus") | (1,611) |
| S7 | S3 OR S5 OR S6 | (727,621) |
| S6 | "nurse" | (272,623) |
| S5 | (MH "Physicians+") OR (MH "Medical Staff, Hospital+") | (123,805) |
| S4 | S1 OR S2 | (136,764) |
| S3 | (MH "Health Personnel+") OR (MH "Allied Health Personnel") | (597,755) |
| S2 | (MH "Health Knowledge") | (33,706) |
| S1 | (MH "Attitude of Health Personnel+") | (104,994) |

**SCOPUS:**

"health professional*” AND  "Coronavirus Infections"  OR  "Middle East Respiratory Syndrome Coronavirus"  OR  "SARS"  OR  "Coronavirus"  AND  "psycological"  OR  "mental health" OR “psychosocial” OR “resilience” OR “wellbeing”

**PSYCHINFO:**

| S16 | ((S1 OR S12 OR S13) AND (S9 OR S10 OR S11)) AND (S2 OR S4 OR S5 OR S6 OR S7 OR S8) | (101) |
| --- | --- | --- |
| S15 | (S1 OR S12 OR S13) AND (S9 OR S10 OR S11) | (39,027) |
| S14 | S1 OR S12 OR S13 | (103,588) |
| S13 | Resilience, Psychological | (15,241) |
| S12 | Adaptation, Psychological | (56,344) |
| S11 | physician | (90,014) |
| S10 | nurse | (69,922) |
| S9 | health personnel | (113,494) |
| S8 | mers | (337) |
| S7 | mers coronavirus | (6) |
| S6 | sars or severe acute respiratory syndrome | (1,317) |
| S5 | h1n1 influenza | (430) |
| S4 | coronavisus infection | (29,410) |
| S2 | Disease Outbreaks | (2,970) |
| S1 | Attitude of Health Personnel | (34,692) |

**ESM 2. Study selection flow-chart. The review included studies from ten different countries, mainly Taiwan, Republic of Singapore, Canada and China. The vast majority of the studies had a cross sectional design and were published between 2004 and 2020.**

**Database last searched on the 24th of April 2020**

- PUBMED: 166
- CINAHL: 210
- SCOPUS: 568
- PsycINFO: 101
- Other sources: 10

Additional records identified through other sources
(n = 10 )

Records identified through database searching
(n =1055)

## Identification

Records after duplicates removed
(n = 691)

## Screening

Records screened
(n =691)

Records excluded after abstract and title reading
(n = 629 )

Full-text articles assessed for eligibility
(n = 62 )

## Eligibility

Full-text articles excluded, based on inclusion and exclusion criteria
(n = 26 )

## Included

Studies included in qualitative synthesis
(n = 36 )

*From:*  Moher D, Liberati A, Tetzlaff J, Altman DG, The PRISMA Group (2009). *P*referred *R*eporting *I*tems for *S*ystematic Reviews and *M*eta-*A*nalyses: The PRISMA Statement. PLoS Med 6(6): e1000097. doi:10.1371/journal.pmed1000097

**ECM 3. Main findings of the included papers.**

| First author, year | Main findings |
| --- | --- |
| *quantitative studies* |  |
| austria-corrales et al., 2011 | The highest proportion of medical residents with burnout syndrome was those in their **second year of specialization** in the area of pneumology. Medical residents **under 30 years** of **age had a higher probability of presenting burnout syndrome**. |
| bai et al., 2004 | Seventeen staff members **(5 %) suffered from an acute stress disorder**; stepwise multiple logistic regression analysis determined that **quarantine was the most related factor**. Sixty-six staff members (**20 %) felt stigmatized and rejected in their neighborhood** because of their hospital work, and 20 of 218 health care workers (**9 %) reported reluctance to work or had considered resignation**. |
| bukhari et al., 2016 | There was a significant difference in the **worry and fear scale** of contracting the MERS-CoV infection between participants who worked in **isolation areas, ICUs, and emergency rooms** (mean: 3.01 ± 1.1) compared to participants who worked in areas that are less likely to admit and have MERS-CoV suspected or positive cases (mean: 2.77 ± 1.1; p = 0.031. **Females** were significantly **more worried and fearful** of contracting the virus compared to males (mean: 2.92 ± 1.1 versus 2.61 ± 1.0, respectively; p = 0.045) |
| chan & chan, 2004 | Approximately **20% of the participants reported post-traumatic stress disorder** (PTSD). Four areas were classified as more important using factor analysis: health and relationship with the family, relationship with friends/colleagues, work and spiritual. The areas for **coping** strategies were **clear directives/precautionary measures, ability to give feedback to/obtain support from management, support from supervisors/colleagues, support from the family, ability to talk to someone and religious convictions**. Support from supervisors/colleagues was a significant negative predictor for psychiatric symptoms and PTSD. **Work and clear communication** of directives/precautionary measures also helped **reduce psychiatric symptoms**. |
| chen et al., 2005 | The results showed that **11% of the nurses** surveyed had **stress reaction syndrome**. The symptoms of psychological stress reactions included **anxiety, depression, hostility, and somatization**. The highest rate of stress reaction syndrome was observed in the group that originally worked in a **high-risk unit**, and the conscripted group experienced the most severe distress on average. |
| chen et al., 2005 (b) | The problem that generated the **most severe stress** in both **physicians and nurses was emergency resuscitation**. The second and third items were **inadequate isolation room** and **inadequate personal protection** in the physicians group, and changes in process of fever patient management and inadequate personal protection in the nurses group. |
| chong et al., 2004 | In the initial phase of the **outbreak**, when the infection was spreading rapidly, **feelings of extreme vulnerability, of extreme uncertainty and threat to life** were perceived, dominated by **somatic and cognitive symptoms** of anxiety. During the ‘**repair’** phase, when the infection was being brought under control, **depression and avoidance were evident**. The estimated prevalence of **psychiatric morbidity** measured by the Chinese Health Questionnaire was about **75%**. |
| chua et al., 2004 | **Stress levels** in the outbreak for both HCWs and healthy control subjects **was approximately 50% more than the normative value**. HCWs appeared to be **protected from stress**, with significantly more positive psychological effects than were observed in control subjects. Such positive responses were reported by 94% (n = 256) of HCWs and included **awareness of hygiene** (85%), **focus on current affairs** (77%), **unity** (51%), and **awareness of danger** (41%).  However, **89%** of HCWs (n = 241) also experienced **negative sequelae such as tiredness** (71%), **worry about health** (59%), and **fearing social contact** (46%). HCWs who **were confident about infection control** (74%, n = 179**) had lower stress levels** and **fewer negative effects**. Confidence about infection control was **independent of education**, perhaps because of 100% attendance at infection-control training. |
| goulia et al., 2010 | **More than half of HCWs** experienced moderately **high levels of worry about the pandemic**, with auxiliary staff presenting the higher degree of worry and nurses being more worried than medical staff.  The **most frequent concern** was **for infection of family and friends and the consequences of the disease on their health**. The degree of worry about the pandemic was an independent correlate of psychological distress.  Few HCWs (6.6%) had restricted their social contacts and fewer (3.8%) felt isolated by their family members and friends because of their hospital work, while a low percentage (4.3%) would take a leave to avoid infection. However, worries and **degree of worry were significantly associated with intentional absenteeism and restriction of social contacts**.  Most HCWs considered that it was not possible to avoid their duties in an emergency situation due to the pandemic and **they would continue working despite the potential risks**. |
| grace et al., 2005 | A significantly **higher rate of psychological distress** was seen among physicians **providing direct care to SARS patients** (45.7%) than among those not providing direct care (17.7%), and physicians providing direct care reported feeling more **stigmatized**. Several physicians (10.9%) reported **entering the hospital despite experiencing identified SARS symptoms**. The most frequent SARS concerns were about the care of non-SARS patients following suspension of nonessential services and loss of physician income. |
| kahlid et al., 2016 | HCWs ethical obligation to their profession pushed them to **continue with their jobs**. The main sentiments centered upon **fear of personal safety and well-being of colleagues and family**. **Positive attitudes in the workplace, clinical improvement of infected colleagues, and stoppage of disease transmission** among HCWs after adopting strict protective measures **alleviated their fear and drove them through the epidemic**. |
| lai et al., 2020 | A considerable proportion of participants reported **symptoms of depression** (634 [50.4%]), **anxiety** (560 [44.6%]), **insomnia** (427 [34.0%]), and **distress** (899 [71.5%]). **Nurses, women, frontline health care workers**, and **those working in Wuhan**, China, reported **more severe degrees** of all measurements of **mental health symptoms** than other health care workers.  Frontline health care workers engaged in direct diagnosis, treatment, and **care of patients with COVID-19** were associated with a **higher risk of symptoms of depression** (OR, 1.52; 95% CI, 1.11-2.09; P = .01), **anxiety** (OR, 1.57; 95% CI, 1.22-2.02; P <0 .001), **insomnia** (OR, 2.97; 95% CI, 1.92-4.60; P < 0.001), and **distress** (OR, 1.60; 95% CI, 1.25-2.04; P <0.001). |
| lee et al. 2005 | **(a) immediate reactions to the mission:** nurses showed mostly a positive attitude, someone felt anxiety, fear, depression and loss of control; **(b) major stressors inherent in caring for SARS patients:** worries about colleagues, patients and family members; frequent changes on infection control measures, lack of knowledge of the virus, lack of adequate protective equipment, discomfort during protective equipment wearing, caring for colleagues who dyed, conflict with doctors, (**c) effective measures to reduce stress;** psychiatric service, Enforcement of stringent infection control procedures, supplies of adequate protective equipment and the regular education program (**d) coping strategies;** adoption of more active personal protective measures, learn more about the disease and engage in health-promoting behaviours, After work debriefing section, recreational activities, chatting with families and friends by phone, avoid watching much news about SARS outbreak, (**e) motivators to join future missions;** having adequate and sufficient protective equipment, followed by clear information about the disease **(f) evaluation of psychiatric services.** the psychiatric services were a great form of support |
| lu et al. 2006 | Twenty-two participants (17.3%) developed significant mental symptoms, and 105 participants (82.7%) showed no obvious symptoms. **A higher level of neuroticism resulted in poorer mental health, and a higher degree of maternal care resulted in better mental health**. |
| lung et al. 2009 *(follow-up lu et al., 2006 study)* | At **one year follow-up**, **15.4% of the participants had mental health symptoms**. Healthcare workers that had mental symptoms at follow-up reported the symptoms were associated with **daily life stress and not the SARS crisis**. The **physicians had more somatic symptoms** than nurses, **suggesting different professions have different impact on mental health**. **Additionally, individual’s early maternal attachment and neuroticism were found to have greater effect on mental health** of life-threatening stress. |
| matsuishi et al. 2012 | **‘Anxiety about infection’ was stronger among younger workers**. Workers in their 50s were more exhausted than younger one. ‘**Feeling of being protected’ appeared to be stronger in middle-aged workers than in younger workers**. Nurses and others were significantly more anxious about infection. **‘Exhaustion’ and ‘workload’ were significantly stronger in nurses than in MDs**. It could be related to the time spent with the infected patients. Hospital workers in **high-risk work environments** felt significantly more ‘**anxiety** about infection’, ‘**exhaustion’** and ‘**workload’** and had significantly **higher symptoms of PTDS** than workers in low-risk work environments. |
| maunder et al. 2004  (follow-up of the 2003 study) | The attitudes questionnaire dimensions were all significantly correlated with PTSD symptoms. **More frequent PTSD symptoms were associated with SARS patient contact** (F(1, 1553) 24.93, p<0.001) **and being a nurse**. Nurses and other healthcare workers who have a **direct contact with SARS patients suffer a more acute traumatic response**. The mediating factors were: i) **fear for one’s health and the health of others**; ii) **social isolation and avoidance**; and iii) **experiencing increased job stress**. Also workload and as being assigned to **unusual task seemed to increase the level of stress**. Another aspect that contribute to increased psychological stress was **social isolation and stigma**. |
| maunder et al. 2006  (follow-up of the 2004 study) | **Toronto healthcare workers showed significantly higher level of burnout, psychological distress and post-traumatic stress**. They were more likely to have reduced patient contact and work hours and to report behavioural consequences of stress. Variance in adverse outcomes was explained by a **protective effect of the perceived adequacy of training and support and by a provocative effect of maladaptive coping style and other individual factors**. In contrast to studies of distress during and shortly after the SARS outbreak, job stress related to conflict, workload, and conscription to new duties did not mediate long-term outcome. However, perceived adequacy of training, moral support, and protection were associated with better outcome. |
| oh et al., 2017 | The **overall stress, professionalism, and nursing intention** scores for the first-hand experience group **were higher than the scores of send-hand experience**. The present study revealed that **prior outbreak nursing experience was importantly associated with an intention to provide care for patients** with a newly emerging infectious disease in the future. In addition, a positive association between nursing intention and professionalism in nursing, but a **negative association between nursing intention and stress was detected**. |
| phua et al. 2005 | The respondents reported significantly more use) **of emotion-focused and problem-focused coping strategies** (i.e., adaptive strategies) than less useful coping strategies (p<0.001. The **physicians** significantly preferred (p<0.001) **humour** as a coping strategy compared with the nurses. **17.7%** of responders scored ≥26 on the IES, indicative of **psychiatric morbidity**. These 17.7% who had psychiatric morbidity reported significantly higher use of all three categories of coping responses compared with those who did not have psychiatric morbidity. Psychiatric morbidity was found in 18.8% of the sample with GH28. **Who had psychiatric morbidity reported significantly higher use of less-useful coping responses** only, compared with those who did not have psychiatric morbidity. **Nurses to reported significantly higher psychiatric morbidity than physicians** (p = 0.03) on the IES but not on the GHQ 28. *The Filipino physicians and nurses*, the majority of whom were Christians, **indicated religion as a coping measure**. Use of **alcohol and drugs never resulted as coping strategy**. |
| sin et al. 2004 | **23.4% of the participants experienced severe psychiatric symptoms and 12.8% showed symptoms of post-traumatic** stress disorder (IES total ≥30). **Coping strategies**: Taking ample precautionary measures reduce the staff’s perceived risk of exposure. **This ‘perceived security’ helped to reduce emotional stress, fear and anxiety at work**. The majority of the responders (80%) reported as a useful strategy that helped them cope with the SARS situation **to have someone to talk to**, whether a family member, supervisor or colleague. In total, **89.4% of the participants felt that SARS information and communication of directives were important in helping them cope with the situation**. **Changes in life’s priorities**: health, relationship with family/friends/colleagues, work and spiritual beliefs were the life priorities more important during the outbreak. |
| styra et al. 2008 | **HCWs who were working in high-risk units experienced greater distress**. Contrary to expectations, HCWs who experienced greater contact with SARS patients while working in the high-risk units were less distressed. Data showed that **caring for only one patient with SARS is significantly more stressful than caring for none or caring for two or more patients with SARS**. The experience of HCWs who cared for several patients with SARS and were exposed on repeated occasions without being infected may have increased their confidence in the management of patient with SARS. |
| su et al. 2007 | No difference between the two groups was found in the **prevalence of post-traumatic stress symptoms**, yet, three unit subjects (SARS ICU, SARS regular and Neurology) had significantly higher rates than those in CCU (29.7% vs. 11.8%, respectively) (p < 0.05).  **For the SARS unit nurses, significant reduction in mood ratings, insomnia rate and perceived negative feelings as well as increasing knowledge and understanding of SARS at the end of the study (all p < 0.001) indicated that a gradual psychological adaptation had occurred**. The adjustment of nurses in the more structured SARS ICU environment, where nurses care for even more severely ill patients, may have been as good or better than that of nurses in the regular SARS unit.  **Occurrence of psychiatric symptoms was linked to direct exposure to SARS patient care, previous mood disorder history, younger age and perceived negative feelings**. **Positive coping attitude and strong social and family support may have protected against acute stress**. |
| tam et al, 2004 | Psychological morbidity = 56.7%. **Variables associated with psychological morbidity**: **female gender, poor self-rated physical health, high level of job-related stress and inadequate support in two aspects**: **counselling and psychological support from the employer, and insurance and compensation**.  **Variables associated with high job-related stress: younger age being nurse, experience of direct care of SARS patients, poorer self-rated physical health condition**. Working in a SARS unit was associated with increased job-related stress but not with psychological morbidity. One of the most distressing aspects was healthcare workers becoming infected. The role factor illustrated the **conflict between the professional responsibility of the healthcare workers and their sense of incompetence**. For the work-related factor, most of the respondents complained about **ambiguity of policies, ineffective dissemination of information and lack of a feedback mechanism**, rather than insufficient protective wear or hazards in the workplace. The frequent changes to infection control policies and restructuring of services exacerbated the sense of uncertainty. **Positive aspects:** epidemic increased awareness of personal and environmental hygiene. Responders reported that they gained a new appreciation of life a deepening of their relationships with family members and colleagues, with the realization of how quickly they could lose them. They also reported making **positive changes to the priorities in their lives**, such as relaxing more and deriving more enjoyment from life. |
| tan et al., 2020 | Sixty-eight (14.5%) participants screened positive for anxiety, 42 (8.9%) for depression, 31 (6.6%) for stress, and 36 (7.7%) for clinical concern of PTSD. **The prevalence of anxiety was higher among nonmedical health care workers than medical personnel** (20.7% versus 10.8%), after adjustment for age, sex, ethnicity, marital status, survey completion date, and presence of comorbid conditions. **Similarly, higher mean DASS-21 anxiety and stress subscale scores and higher IES-R total and subscale scores were observed in nonmedical health care workers.** |
| tham et al. 2005 | Frontline healthcare workers **suﬀered from signiﬁcant stress from various sources during the SARS outbreak**. The most prevalent of these were hazards to personal health, role conﬂicts and frequent changes to infection control policies. The healthcare workers’ psychological morbidity was best understood by their perceptions of personal vulnerability, stress and support in the workplace. Although the outbreak was tragic and frightening, many healthcare workers perceived some beneﬁts and personal growth in the struggle with the disaster. |
| tolomiczenko et al. 2005 | While all groups found SARS stressful, **nurses reported a greater impact on morale and job satisfaction**. Nurses **relied more on peer support than doctors, felt less informed and less involved in decision-making than doctors**, and were more likely to report that infection control procedures were not strict enough. |
| verma et al. 2004 | **Those GPs who were directly involved in the care of patients with SARS were significantly more likely to be GHQ case as compared to those not involved in the care of patients with SARS** (P = 0.02; OR = 2.9; 95% CI, 1.3-6.3). The mean score of the GHQ somatic, anxiety and social dysfunction subscales were significantly higher in GPs as compared to TCM Practitioners (P <0.001). The GHQ total score as well as the subscales was significantly correlated with the IES-R and stigma subscales. |
| wong et al. 2005 | **SARS had caused a significant level of distress among staff working in the Emergency Department**. The distress level was highest for nurses, followed by doctors and HCA. **The three most important variables that could account for the distress level were loss of control/vulnerability, fear for self-health, and spread of the virus**. Overall, the more frequently adopted coping strategies were acceptance, active coping, and positive framing |
| wu et al. 2009 | About 10% of the respondents had experienced high levels of posttraumatic stress disorder (PTSD) symptoms since the SARS outbreak**. Respondents who had been quarantined, or worked in high-risk locations such as SARS wards, or had** **friends or close relatives who contracted SARS, were 2 to 3 times more likely to have high PTS symptom levels, than those without these exposures.** Respondents’ perceptions of SARS-related risks were significantly positively associated with PTSD symptom levels and partially mediated the effects of exposure. **Altruistic acceptance of work-related risks was negatively related to PTS levels.** |
| *qualitative studies* |  |
| almutairi et al., 2018 | Within the participants’ rich and illuminating experiences, four themes were identified: caring for others in the defining moments, perceived prejudice behaviours and stigmatization, lived moments of traumatic fear and despair, and denial and underestimation of the seriousness of the disease at the individual and organizational levels. Survivors still suffered as a result of their traumatic experiences, which might negatively influence their performance. As these survivors are vulnerable, it is their organization’s responsibility to provide a system that embraces HCPs during and after disastrous events. |
| corley et al., 2010 | Eight common themes emerged: the wearing of personal protective equipment; infection control procedures; the fear of contracting and transmitting the disease; adequate staffing levels within the intensive care unit; new roles for staff; morale levels; education regarding extracorporeal membrane oxygenation; and the challenges of patient care. |
| holroyd & mcnaught, 2008 | Six themes were identified, with related subthemes: 1 The suddenness of SARS (Panic and uncertainty, Unpreparedness, Death of co-workers), 2. Impacts on professional nursing practice (Training and deployment, Confusion about infection control measures, Adapting to change, Nurse–patient relationships, Professional commitment under strain) 3. Personal impacts (Fearing widespread infection, Fearing self-infection Developing psychological symptoms), 4. Community and families (Avoiding contact, Social isolation, Meeting family responsibilities, Valuing family and friends, Changing social practices), 5. Community and cultural responses (Seeking alternative remedies, Blaming healthcare workers), 6. Being prepared (Changing healthcare policy, Remaining vigilant). |
| khee et al. 2004 | Two mail trends: 1 trend: dynamic development of specific behaviours, 2 trend development of serious issue when some specific event had occurred, such as the death of a colleague. Prevailing issues:1, fear, 2 vigilance, 3 detachment, 4 separation anxiety, 5 mortality concerns, 6 death, 7 deprivation, 8 disruption, 9 discrimination, 10 sense of duty, 11 discrepancy, 12 frustration, 13 Sense of Unworthiness, 14 deja vù, 15 abnormal behaviours, 16 disclosure. Positive feedback: 1 broadcast of information, 2 dynamic development, 3 sense of duty, 4 team spirit, 5 self-sacrifice, 6 divine intervention, 7 reliable support, 8 Overcoming negative thoughts, 9 delayed emotion, 10 positive feedback. |
| kim et al. 2018 | Five themes were identified: “Going into a dangerous field,” “Strong pressure because of MERS-CoV,” “The strength that make me endure,” “Growth as a nurse,” and “Remaining task.” |
| maunder et al. 2003 | Staff affected by fear of contagion and of infecting family, friends and colleagues. Caring for health care workers as patients and colleagues was emotionally difficult. Uncertainty and stigmatization were prominent themes for both staff and patients |

**ECM 4. The characteristics of the included studies.**

| ***Variable*** | ***Variable operazionalization, numbers (percentage)*** |
| --- | --- |
| **Country/City** | - Taiwan, 8 studies (22^.^2%) - Republic of Singapore, 7 Studies (19^.^4%) - Canada, Toronto 6 studies (16^.^7%) - China, 6 studies, (Hong Kong=4; Beijing=1, multisite=1) (16^.^7%) - Saudi Arabia, 3 studies (8^.^3%) - South Korea, 2 studies (5^.^5%) - Greece, Ioannina, 1 study (2^.^8%) - Australia, Queensland, 1 study (2^.^8%) - Japan, 1 study (2^.^8%) - Mexico, Mexico city, 1 study (2^.^8%) |
| **Setting** | - General hospitals/general teaching/university hospital,15 studies (41^.^7%) - Tertiary care hospitals, 7 studies (19^.^4%) - Emergency department/ ICU, 4 studies (11^.^1%) - Community/ public/ local hospital, 3 studies (8^.^3%) - SARS unit, 1 study (2^.^8%) - Psychiatric services/department, 1 study (2^.^8%) - Rehabilitation service/department, 1 study (2^.^8%) - University, 1 study (2^.^8%) - Not stated, 3 studies (8^.^3%) |
|  |  |
| **Profession** | - Nurses, 28 studies (77^.^8%) - Physicians, 23 studies (63^.^9%) - HCWs (not specified), 11 studies (30^.^5%) - Supportive staff (Allied health workers/healthcare assistants   Auxiliary staff), 5 studies (13^.^9%)   - Administrative staff/management, 5 studies (13^.^9%) - Respiratory therapists, 3 studies (8^.^3%) - Occupational therapists, 3 studies (8^.^3%) - Physiotherapist, 3 studies (8^.^3%) - Clerical staff, 3 studies (8^.^3%) - Speech therapists, 2 studies (5^.^5%) - Technicians, 2 studies (5^.^5%) - Pharmacists, 1 study (2^.^8%) - Traditional Chinese medicine practitioner, 1 study (2^.^8%) - Laboratory staff, 1 study (2^.^8%) |
| **Study participants’ numbers** | |
| **Range in number of respondents** | Qualitative studies: from 7 to 188  Quantitative studies: from 26 to 1625 |
| **Total respondents** | Qualitative studies: N=246  Quantitative studies: N=13711 |

**ESM 5. Summary of preventive strategies for psychosocial distress.**

| **Type of strategy** | **Description of the strategy** | **Study** |
| --- | --- | --- |
| **Policy related strategy** | Develop a strategic plan for future outbreak. | Corley et al, 2010; Holroyd and McNaught, 2008; Kim et al, 2018; Lu et al, 2006; Lung et al, 2009;  Maunder et al, 2006; Sin & Huak, 2004;  Tolomiczenko et al, 2005; Wong et al, 2005; |
|  | Develop public campaigns to protect HCWs*. | Matsuishi et al, 2012 |
| **Organization-related strategy** | Offer favourable work conditions | Austria-Corrales et al, 2011; Bai et al, 2004 Matsuishi et al, 2012; Maunder et al, 2006 Su et al, 2007 |
|  | Provide HCWs with all the Personal Protective Equipment (PPE) necessary to work safely and reduce their risk. | Chen et al, 2005a, 2005b Goulia et al, 2010 Kahlid et al, 2016 |
|  | Recognize HCWs efforts, and provide positive feedback. | Kahlid et al, 2016; Maunder et al, 2006 |
|  | Promote personal coping strategies (such as altruism, acceptance, resilience, humor) | Lee et al, 2005; Wong et al, 2005; Wu et al, 2009 |
| **Person-directed strategy** | | |
| **Practical** | Provide HCWs accurate and timely information to reduce uncertainty. | Bai et al, 2004; Corley et al, 2010; Goulia et al, 2010; Kahlid et al, 2016; Matsuishi et al, 2012;  Maunder et al, 2003, 2004, 2006; Sin & Huak, 2004 |
|  | Provide HCWs training about how to protect their-self and dealing with the infected patients. | Bai et al, 2004; Bukhari et al., 2016; Chen et al, 2005a, 2005b; Chua et al, 2004; Corley et al, 2010;  Maunder et al, 2003, 2004, 2006; Oh et al, 2017;  Sin & Huak, 2004 |
| **Personal** | Provide psychological support before, during and after the outbreak | Almutairi et al, 2018; Bai et al, 2004; Chan and Chan, 2004 ; Chen et al,2005a, 2005b; Chong et al, 2004; Corley et al, 2010; Grace et al, 2005; Khee et al, 2004; Kim et al, 2018; Lai et al, 2020; Lee et al, 2005; Matsuishi et al, 2012; Phua et al, 2005; Styra et al, 2008; Su et al, 2007; Tam et al, 2004; Tan et al, 2020; Tham et al, 2005; Verma et al,2004; Wong et al, 2005 ; Wu et al, 2009 |
|  |  |  |
|  | Promote social mutual support within the HCWs team, reduce conflict and reduce the effect of social isolation. | Khee et al, 2004; Lee et al, 2005; Maunder et al, 2003, 2004, 2006 |
|  | Provide social support for HCWs family. | Bukhari et al, 2016; Grace et al, 2005 |
|  | Provide physical support | Bai et al, 2004; Goulia et al, 2010; Maunder et al, 2003 |
|  |  |  |

** HCWs= health care workers*
